# Supplementary material for: Comparative evaluation of the sleep quality metrics between a cardboard bed and a camp cot: a randomized controlled crossover study
Source: PeerJ. 2024 May 24;12:e17392. doi: 10.7717/peerj.17392 (PMC11129694; doi:10.7717/peerj.17392)
Supplement: Supplemental Information 3 [file peerj-12-17392-s003.pdf]

Confirmation of Participant Eligibility

質問紙/ Questionnaire

＜Japanese / English＞

調査日＿＿＿＿年＿＿＿＿月＿＿＿＿日/ Date/Month/Year

年齢：＿＿＿歳/Age.＿＿＿y

ID:＿＿＿＿＿.　性別　：男性　・　女性./ Gender; Male. Female

BMI：＿＿＿＿＿＿.

BMI ＝ 体重(kg) /身長(m)^{2}として計算します。
BMI = measured weight (kg) / height^2 (m)

平日の平均的な就寝時間と起床時間／Average bedtime and wake-up time on weekdays

就寝時間／Bedtime＿＿＿＿＿＿　　起床時間／Wake-up time＿＿＿＿＿＿.

本研究では以下に該当する方は研究にご参加いただくことができません。該当しないかご確認ください
Please check if any of them apply to you.

☐ 以下の状況に該当しません/ Not applicable

☐ （番号:　　）に該当します/Applicable (No.　　　)

1：継続する不眠など睡眠に関連した問題がありますか？
Do you experience ongoing insomnia or any other sleep-related issues?

2：精神や睡眠に影響を及ぼす薬剤を服用されていますか？
Are you taking any medication that affects your mental state or sleep?
治療中の病名または服薬中の薬剤名＿＿＿＿＿＿＿＿＿＿＿＿＿.

3：夜勤または交代勤務に従事していますか？
Are you engaged in night shifts or rotating shifts?

4. 実験期間内に3時間以上の時差がある地域へ渡航予定ですか？
Do you have plans to travel to a region with a time difference of more than three hours?

5. 妊娠中(妊娠の可能性ありを含む)および授乳中ですか？
Are you pregnant or breastfeeding?

6. 急変の可能性のある疾病にかかっていますか？
Do you have a disease with a potential for rapid change?
病名＿＿＿＿＿＿＿＿＿＿.

7. 睡眠を妨げるような身体症状（疼痛・掻痒感・頻尿等）を有していますか？
Do you have any physical symptoms that interfere with sleep, such as pain, itching, or frequent urination?

8. 現在喫煙されていますか？
Are you currently smoking?

Pre-experiment (Before Bedtime) Survey

＜Japanese / English＞

質問紙/ Questionnaire

ID:________.　　調査日______年______月______日/ Date/Month/Year

☐ キャンプコット／Camp cot　☐段ボールベッド／Cardboard bed

直近3日間の睡眠状況についてお答えください。
Please describe your sleep pattern in the past three days.

	就寝時間 Bedtime	起床時間 Wake-up time	睡眠の問題／Sleep Problems
3日前 Three nights ago			☐なかなか寝付けなかった／Struggled to fall asleep<br>☐何度も目が覚めた:／Frequently woke up during the night<br>☐予定より早く目が覚めた／Woke up earlier than intended<br>☐日中すごく眠たかった／Felt strong daytime sleepiness
2日前 Two nights ago			☐なかなか寝付けなかった／Struggled to fall asleep<br>☐何度も目が覚めた:／Frequently woke up during the night<br>☐予定より早く目が覚めた／Woke up earlier than intended<br>☐日中すごく眠たかった／Felt strong daytime sleepiness
1日前 Last night			☐なかなか寝付けなかった／Struggled to fall asleep<br>☐何度も目が覚めた:／Frequently woke up during the night<br>☐予定より早く目が覚めた／Woke up earlier than intended<br>☐日中すごく眠たかった／Felt strong daytime sleepiness

本日の体調で当てはまるものをお選びください（複数回答可）
Please select all that apply regarding your condition today (multiple responses allowed)

☐コロナを疑わせる症状（発熱・倦怠感・咳・鼻汁・咽頭痛等）
Symptoms suggestive of COVID-19 (fever, fatigue, cough, runny nose, sore throat, etc.)

☐生活に影響を及ぼす強い疲労感／Severe fatigue affecting daily life

☐生活に影響を及ぼす強い不安やストレス／Severe anxiety or stress affecting daily life

☐首や肩の酷い凝りや痛み／Severe stiffness or pain in the neck or shoulders

☐背中や肩の酷い凝りや痛み／Severe stiffness or pain in the back or shoulders

☐その他睡眠に影響を及ぼしそうな体調不良／Other health issues likely to affect sleep

☐特になし／None

下記に該当する場合は、チェックをつけてください／ Please check the box if the following applies.

☐本日、飲酒はされましたか？
Have you had any alcoholic drinks today？

☐夕方5時以降、カフェインを含む飲み物（コーヒー・紅茶・緑茶等）は飲まれましたか？
Have you had any caffeinated beverages after 5 PM?

☐本日、何らかの薬剤を服用されましたか？（スタッフに飲んだお薬の名称を伝えてください）
Are you taking any medication today?

☐（女性の方のみ）本日は月経中あるいは月経予定日から1週間以内ではありませんか？
※月経予定日がわからない場合には、最終月経終了後2週間を過ぎていませんか？

Post-Wakeup Survey

＜Japanese / English＞

質問紙/ Questionnaire

調査日_______年_______月_______日/ Date/Month/Year

ID:________.

以下の質問にお答えいただき、該当する数字を選んで〇をつけてください。
Please answer the following question and mark the corresponding number with a circle.

1.ベッドの寝心地はどうでしたか？
How was the comfort of the bed?

考えうる最高の寝心地<br>The best possible sleeping comfort	0	1	2	3	4	5	6	7	8	9	10	考えうる最悪の寝心地<br>The worst possible sleeping comfort

2.ベッドの硬さはどうでしたか？
How was the firmness of the bed?

考えうる最も柔らかい寝床<br>The softest imaginable	0	1	2	3	4	5	6	7	8	9	10	考えうる最も硬い寝床<br>The The firmest imaginable

3.首から肩にかけての凝りや痛みの程度をお答えください。
How would you rate your cervical stiffness

全く感じない<br>None	0	1	2	3	4	5	6	7	8	9	10	考えうる最大の凝りや痛み<br>The maximum conceivable stiffness

4.背中から腰にかけて感じる凝りや痛みの程度をお答えください。
How would you rate your lumbar stiffness

全く感じない<br>None	0	1	2	3	4	5	6	7	8	9	10	考えうる最大の凝りや痛み<br>The maximum conceivable stiffness

5.昨夜はどの程度よく眠れましたか？
How satisfied were you with your sleep last night?

非常によく眠れた<br>Extremely satisfied	0	1	2	3	4	5	6	7	8	9	10	全く眠れなかった<br>Not satisfied at all

6. 現在の眠気の程度についてお答えください
How sleepy do you feel right now?

1	2	3	4	5	6	7	8	9
非常にはっきり目覚めている<br>Extremely alert		目覚めている<br>Alert		どちらでもない<br>Neither alert nor sleepy		眠い<br>Sleepy, but no difficulty remaining awake		とても眠い（眠気と戦っている）<br>Extremely sleepy, fighting sleep
